# Comprehensive and Highly Accurate Measurements of Crane Runways, Profiles and Fastenings

**DOI:** 10.3390/s17051118

**Published:** 2017-05-13

**Authors:** Dirk Dennig, Johannes Bureick, Johannes Link, Dmitri Diener, Christian Hesse, Ingo Neumann

**Affiliations:** 1 Geodetic Institute, Leibniz Universität Hannover, Nienburger Str. 1, 30167 Hannover, Germany; bureick@gih.uni-hannover.de (J.B.); link@gih.uni-hannover.de (J.L.); diener@gih.uni-hannover.de (D.D.); neumann@gih.uni-hannover.de (I.N.); 2 Dr. Hesse und Partner Ingenieure, Veritaskai 6, 21079 Hamburg, Germany; ch@dhpi.com

**Keywords:** crane runway survey, multi sensor system, cameras, profile laser scanner, calibration

## Abstract

The process of surveying crane runways has been continually refined due to the competitive situation, modern surveying instruments, additional sensors, accessories and evaluation procedures. Guidelines, such as the International Organization for Standardization (ISO) 12488-1, define target values that must be determined by survey. For a crane runway these are for example the span, the position and height of the rails. The process has to be objective and reproducible. However, common processes of surveying crane runways do not meet these requirements sufficiently. The evaluation of the protocols, ideally by an expert, requires many years of experience. Additionally, the recording of crucial parameters, e.g., the wear of the rail, or the condition of the rail fastening and rail joints, is not regulated and for that reason are often not considered during the measurement. To solve this deficit the Advanced Rail Track Inspection System (ARTIS) was developed. ARTIS is used to measure the 3D position of crane rails, the cross-section of the crane rails, joints and, for the first time, the (crane-rail) fastenings. The system consists of a monitoring vehicle and an external tracking sensor. It makes kinematic observations with the tracking sensor from outside the rail run, e.g., the floor of an overhead crane runway, possible. In this paper we present stages of the development process of ARTIS, new target values, calibration of sensors and results of a test measurement.

## 1. Introduction

Crane systems are an important part of the transport and logistics process. In the case of rail-bound cranes, a correctly oriented runway is a prerequisite for good performance, quietness and low wear of wheels and rails. Previous surveys of crane runways aim at determining the position and height of a rail. From these measurements further target values (e.g., gauge) can be determined. The latter are defined in details in guidelines (e.g., in [1]). Their determination requires an agreement between the client, the crane supplier and the crane operator. A survey can be performed in case rails are built or repaired, e.g., in the course of an exchange or maintenance and also when it is necessary to clarify a situation.

The classical method of a crane runway survey consists of an alignment by means of a theodolite and geometric leveling. An optimization of the alignment was achieved by using a laser instead of a theodolite. At one end of the crane a laser was mounted and levelled. It projected a point on the target board, which was initially traversed manually on a vehicle along the rail. The position on the target panel could be read at defined distances in position and height [2]. The process has been automated over time by remote control of the vehicle and digital registration of the deviations of the laser beam.

**Figure 1:**
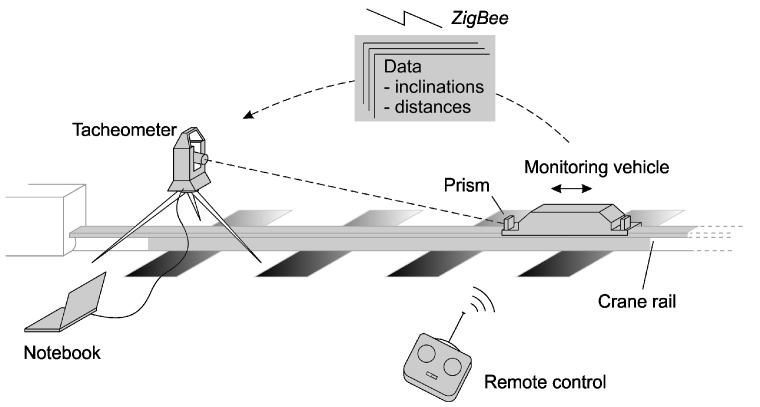
Schematic principle and overview of the components of the crane track surveying system (CTSS) rail control [5]. (Reproduced with permission from Mr. Gerold Olbrich, editorial office, AVN; published by Wichmann Verlag, VDE VERLAG GMBH, 2011.)

With the availability of highly accurate industrial total stations, the 3D position of the rail can be determined eccentrically in the static case [3]. Marjetič et al. developed a simple tool to improve the accuracy of the height determination with total stations [4]. Nevertheless, a major drawback is that each position has to be measured manually. The process is automated by moving a self-propelled vehicle on the rail. Authors of [5,6] compared the self-propelled systems laser measuring system LMS by DEMAG, RailQ^TM^ by Konecranes and RailControl by ThyssenKrupp GfT Gleistechnik and Hanack und Partner. To our knowledge no further substantial developments have been published since then. By integrating an inclinometer, which measures the inclination transversely to the axis of the rail, the offset caused by the inclination of the reflector can be corrected [5]. Depending on the vehicle speed and the recording frequency of the total station, the distance between the measured points are in the range of a few centimeters to a few decimeters. Thus the path of the rail can be described in detail. In case of a kinematic measurement, the total station, as in the case of the alignment, has to observe the prism mounted on the vehicle in the longitudinal direction of the rails (Figure 1).

In case of a determination from the ground, it has to be ensured that the prism is always aligned towards the total station. The use of 360º prisms appears to be suboptimal in light of the findings in [7], where, depending on the design of the prism, possible inaccuracies of up to 2 mm were found.

On a crane runway, static and dynamic forces are at work. During a run of a hall crane the forces pass from the wheel into the rail, horizontally into the rail fastening, and finally into the ground. The situation is similar for cranes running on the ground. The authors of [5] proposed attaching the vehicle to the crane and tracking it kinematically during the run of the crane. The comparison of the measuring results reveals the differences between a crane runway in static state and under load. By attaching an action cam to the vehicle such that it faces the rail, the condition of the rail can be documented with little effort. However, without a spatial reference, an interpretation with respect to concrete stationing is very time-consuming.

**Figure 2:**
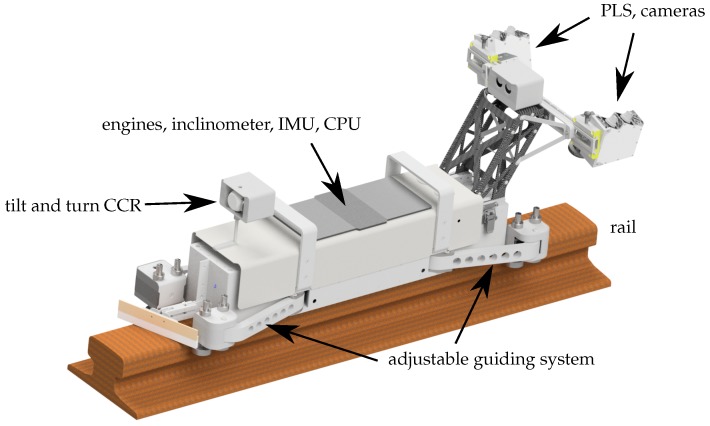
The vehicle of the Advanced Railtrack Inspection System (ARTIS), fully equipped and standing on a crane rail. CCR: corner cube retroreflector; IMU: inertial measuring unit; PLS: profile laser scanner; CPU: central processing unit.

In order to improve the whole process of the crane runway surveying, the new, patented, modular Advanced Railtrack Inspection System (ARTIS, Figure 2) has been developed by the surveying office Dr. Hesse und Partner Ingenieure and the Geodetic Institute of the Gottfried Wilhelm Leibniz Universität Hannover. ARTIS is used to measure the 3D position of crane rails, the cross-section of the crane rail and, for the first time, (crane-rail) fastenings and rail joints.

The paper is structured as follows: In Section 2 new target values are defined and the need for their determination is explained. Section 3 comprises the concept and realization of ARTIS. In Section 4 we evaluate the uncertainties of the process and derive the requirements for the sensor technology. In Section 5 the hard- and software of ARTIS are described. Section 6 depicts the calibration process of ARTIS. The Section 7 shows results of a test measurement. Finally, conclusions are drawn in Section 8.

## 2. Definition of New Target Values for Crane Runways

By now established target values describe the position and height of the rails absolutely and relative to each other, the inclination of the rails, and the inclination difference of opposite rails (e.g., [1]).

For the objective description of the actual state of a crane runway it is necessary to define additional new target values.

### 2.1. Theoretical and Practical Rail Axis

**Figure 3:**
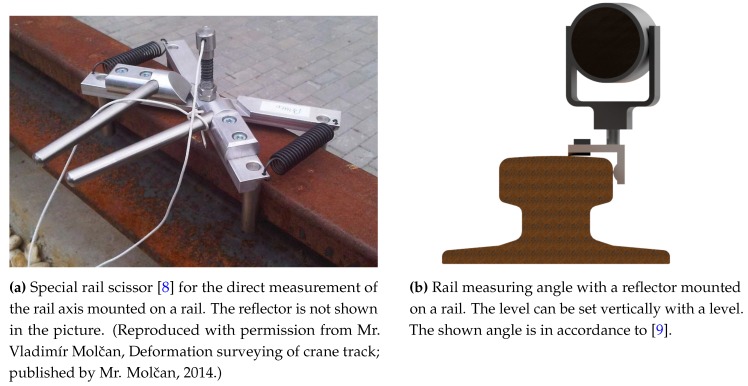
Tools for direct (**a**) and indirect (**b**) determination of rail axes. With the latter, the rail flange can be measured directly.

Target values listed in guidelines (e.g., [1]) refer to the rail axis. Depending on the equipment, they can be determined directly (Figure 3a) or indirectly (Figure 3b). In the former case the direct measure is falsified in a coldly deformed rail head. In the case of the latter, the points are determined in relation to the rail edge and then corrected by an offset. With both methods, it is difficult to take the wear condition of the rail head into account. The condition may change along the rail and as a consequence, the rail axis changes as well. The problem becomes even more severe when the wear of the rail head changes side due to a wandering spur. This often leads to misunderstandings and misinterpretations.

**Figure 4:**
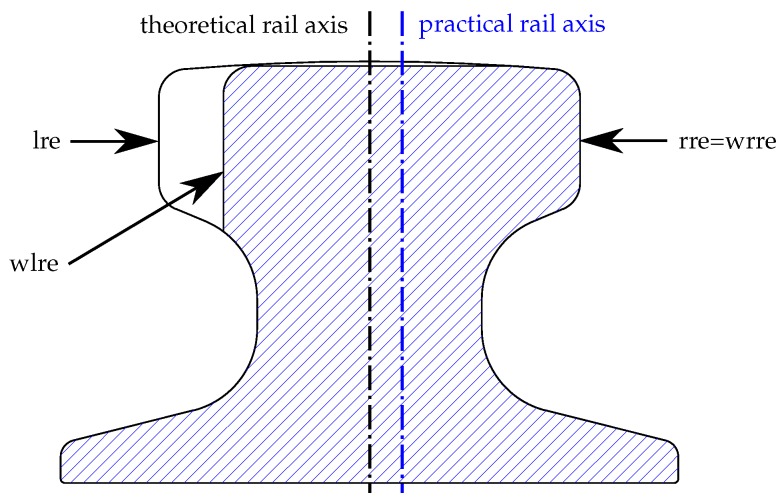
Cross-section of a worn crane rail to illustrate the theoretical and practical rail axis. *lre*: left rail edge; *wlre*: worn left rail edge; *rre*: right rail edge; *wrre*: worn right rail edge.

Clarity is obtained when the actual rail head cross-section is measured consistently. At this point we introduce the concepts of the theoretical and practical rail axes (Figure 4). The practical rail axis constitutes the axis of the worn rail, whereas the theoretical rail axis is the axis of the unused rail head.

According to the four defined edges shown in Figure 4, namely the left rail edge (*lre*), the right rail edge (*rre*), the worn left rail edge (*wlre*), and the worn right rail edge (*wrre*), the distance of the theoretical axis to any of the edges can be calculated according to Equation (1), and the distance of the practical axis to any edge can be calculated according to Equation (2):

(1) $$d_{theoretical\;rail\;axis}:=\frac{\left(rre-lre\right)}{2}$$

(2) $$d_{practical\;rail\;axis}:=\frac{\left(wrre-wlre\right)}{2}$$

In the depicted example in Figure 4 the rail is solely worn at the left edge. The edges “*rre*” and “*wrre*” are located identically.

### 2.2. Defects of the Rail Profile

Rail profiles can have different defects. It is possible to distinguish between defects which are visually recognizable and those which are located below the surface. The following are examples of two errors whose causes are different.

**Figure 5:**
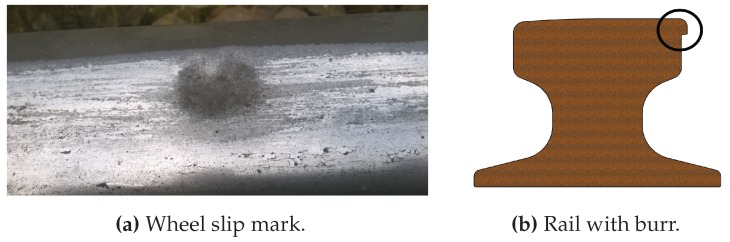
(**a**,**b**) show possible wear of rail profiles.

The wear of the rail profile (Figure 5) can have several causes: excessive loads, poor alignment of the crane runway, unbalanced loading by the crane (e.g., by exceeding tolerances according to International Organization for Standardization (ISO) 12488-1 [1]) or wrong material pairing between the wheel and the rail. The consequences can be plastic deformation or material deterioration at the rail head. As a result of the change in the cross-section, the possible static absorption load changes, which may cause a rail break or a change in the tracking. Another negative effect is the additional stress on the structure. The width of the head, the rail height, the width of the burr (cold rolled lip) the side of the rail head on which the burr is located, cracks as well as breaking out in the rail head, and wheel slip marks can be defined as the target values.

### 2.3. Rail Joints

**Figure 6:**
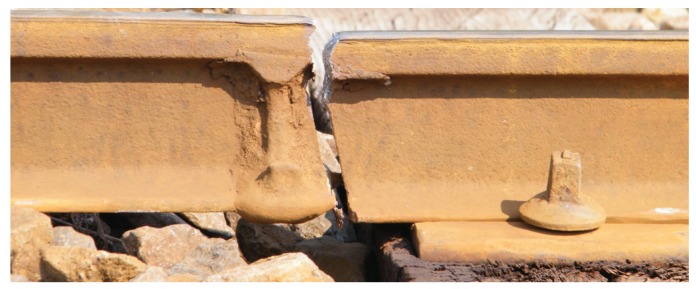
Broken rail joint [10]. (The image is licensed under the Creative Commons Attribution-Share Alike 3.0 Unported license.)

Depending on the machining of the rail ends, the connection between adjoining rails and the ball tolerances, there may be discontinuities at the rail joints (Figure 6). Therefore, their position and design (angular impact, $90^{\circ }$ impact, step impact, connection by welding or tapping, etc.) are of interest.

### 2.4. Rail Fastenings

**Figure 7:**
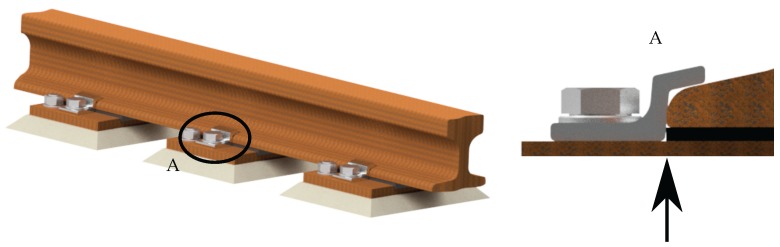
Crane rail with correctly positioned rail fastenings.

For the operation of a crane runway, the knowledge of the position and the condition of the rail fastening (Figure 7) is essential. If the rail fastenings are not installed properly, they could be incorrectly attached to the rail foot or the connections could come loose. As a consequence, the profile is not secured against horizontal movements. During operations, the clearance can amplify and subsequently the movements increase as well. Further consequences may be an increased wear, a failure of the system and in the worst case an accident with personal injuries.

### 2.5. Measurement in the Loaded and Unloaded State

A comprehensive assessment of a crane runway involves the knowledge of its behavior in the loaded and unloaded state. A crane runway which is measured in the unloaded state can have a significantly different position in comparison to the loaded state. This knowledge is a prerequisite to explaining abrasion and utilization reliably. For this reason, the target values related to the position and height (e.g., in [1]) must be determined for both states (see also [5]).

## 3. Concept and Realization

Self propelled rail vehicles have been well-known for many years (e.g., [5]). They are used to transport at least a reflector. If equipped also with an inclinometer, they can provide the inclination of the rail as required by the guidelines (e.g., in [1]), and the inclination value can be used to correct the offset of the reflector. Additional sensors are required for the determination of the new target values described in the previous Section 2. ARTIS is equipped with the following sensors (see also Figure 2):

- 2 profile laser scanner (PLS)
- 2 cameras
- 1 inertial measuring unit (IMU)
- 1 inclinometer
- 2 odometer

The recordings of the cameras in connection with the data of the PLS document the actual status of the profile, of the rail joints, and of the fastenings. Among others, the IMU is used to detect the position of the rail joints. The inclinometer measures the inclination of the rails in the longitudinal and transverse directions. The two odometers measure the distance traveled.

**Figure 8:**
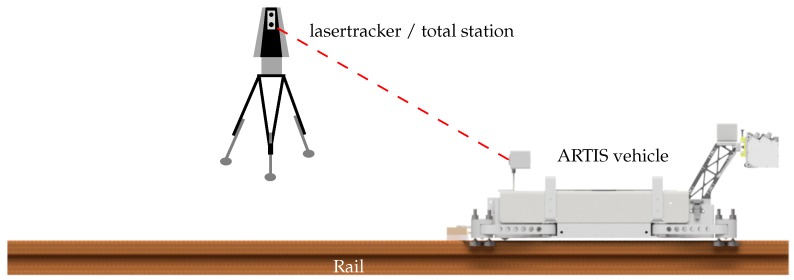
Surveying instrument and the Advanced Railtrack Inspection System (ARTIS) vehicle on a rail.

The PLS and the cameras can be mounted or disassembled on a modular basis depending on the task:

1. When the target values, defined by existing standards, are determined, the vehicle platform (without PLS and cameras) is tracked by means of a total station or a lasertracker. For this purpose, a corner cube retroreflector (CCR) is attached to the vehicle.
2. When all of the previously mentioned target values (see Section 2) are determined, the vehicle platform is equipped with the PLS and cameras. Again, the vehicle, specifically the CCR, is tracked by a total station or a lasertracker. This constellation is depicted in Figure 8.
3. For documentation of the condition of the rail, of the rail fastening and the rail inclination, the vehicle platform is equipped with PLS and cameras and the travelled distance is determined by means of the odometers. A total station or lasertracker is not used.

## 4. Measurement Uncertainty of the Target Variables and Requirements for the Sensor Technology

In case of an eccentric, kinematic measurement, the use of a highly accurate lasertracker, measuring with a high sampling rate is necessary. The lasertracker continuously tracks the position of the vehicle. The target values listed in Table 1 can be determined in combination with further sensors. The data refer to a distance of up to approximately 100 m of measured rail.

**Figure 9:**
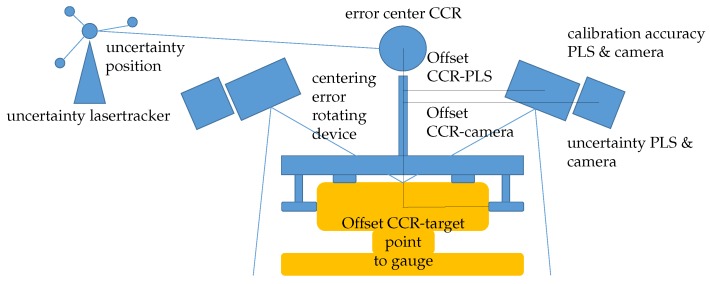
Selection of influencing values for the calculation of the measurement uncertainties.

In order to meet the requirements concerning the accuracies of the target values listed in Table 1, the use of PLS is necessary and adequate, due to their high measuring accuracy and frequency. For the calculation of the measurement uncertainty of the target values, all relevant influencing values must be considered (Figure 9). In the following section, the measurement uncertainties of two target values are considered. These are the position of the rail (measurement with lasertracker, without PLS, absolute reference) and the burr of the rail edges (measurement with PLS, relative reference). They differ in the way that the first target value has to be determined absolutely in the superior coordinate system, whereas for the second one it is sufficient to determine it relatively.

### 4.1. Measurement Uncertainty of the Position of the ARTIS Vehicle

The calculation of the measurement uncertainty of the Y component of the position of the ARTIS vehicle is influenced by the related sensors. Table 2 describes the main influencing values, their measurement uncertainty and the type of the quantification of the uncertainty. According to [11] uncertainties can be distinguished in type A, computed by means of statistical methods, and type B, determined in another way (e.g., information provided by the company or literature). In our case all uncertainties of type A are estimated through an adjustment. All uncertainties of type B result from information provided by the companies. All displayed influencing values are normally distributed respectively assumed to be normally distributed.

Accuracy specifications of the manufacturers specifying an maximum permissable error (MPE) as an upper and lower limit ($a_{+}$ and $a_{-}$) must be converted into a standard measurement uncertainty $u(x_{i})$. Assuming a uniform distribution, the conversion is carried out with Equation (3) according to [11]:

(3) $$u\left(x_{i}\right)=a\;/\;\sqrt{3}\;\mathrm{with}\;a=\left(a_{+}-a_{-}\right)\;/\;2$$

The uncertainties in the following table are all given as standard measurement uncertainties:

Equation (4) describes the schematic, functional relationship in the determination of the *Y* component of the rail axis (pointing in lateral direction of the rail):

(4) $$\begin{array}{cc} Y_{Axis}= & f(Y_{P},z,S_{d},t,Y_{gauge\_line},rot\_dev,CCR_{EC},CCR_{ESS},Z_{CCR-ToR},in)\\ = & Y_{P}+\sin z\cdot S_{d}\cdot \cos t+Y_{gauge\_line}\cdot \cos in+rot\_dev\\ & +CCR_{EC}+CCR_{ESS}+Z_{CCR-ToR}\cdot \sin in \end{array}$$

The measurement uncertainty of the *Y* component of the target point is calculated by setting up the partial derivatives of Equation (4) with respect to the influencing values:

(5) $$\frac{\partial f}{\partial i}\;,i:\mathrm{influencing}\;\mathrm{value}.$$

By insertion into the uncertainty–propagation law Equation (6) follows:

(6) $$\begin{array}{cc} u_{Y_{Axis}}^{2}= & u_{Y_{P}}^{2}-\sin^{2}t\cdot \sin^{2}z\cdot {\left(u_{afix}+u_{avar}\cdot S_{d}\right)}^{2}+\sin^{2}t\cdot \cos^{2}z\cdot {\left(u_{dfix}+u_{dvar}\cdot S_{d}\right)}^{2}\\ & +\cos^{2}z\cdot cos^{2}t\cdot {\left(u_{afix}+u_{avar}\cdot S_{d}\right)}^{2}+u_{gauge\_line}^{2}\cdot \cos^{2}in\\ & +Y_{gauge\_line}^{2}\cdot \sin^{2}in\cdot u_{in}^{2}+u_{rot\_dev}^{2}+u_{CCR_{EC}}^{2}\\ & +u_{CCR_{ESS}}^{2}+\sin^{2}in\cdot u_{Z_{CCR-ToR}}^{2}+Z_{CCR-ToR}^{2}\cdot \cos^{2}in\cdot u_{in}^{2} \end{array}$$

**Figure 10:**
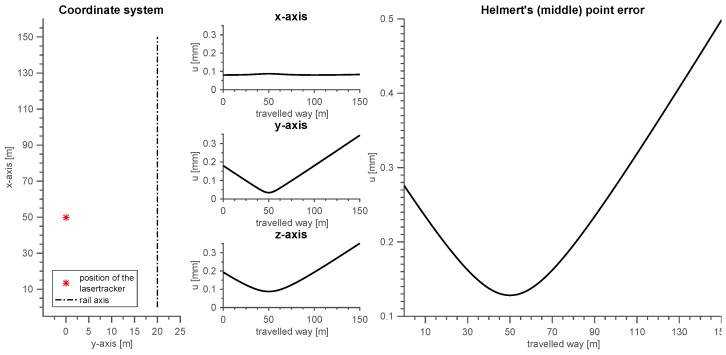
Uncertainties for the *x*, *y* and *z*-axis and Helmert’s point error for the position of the ARTIS vehicle.

The achievable measurement uncertainties are calculated based on exemplary values. The setting assumes a runway track which is 150 m long and 10 m high. The lasertracker is positioned 50 m away from the beginning of the rail, 1.5 m above ground and 20 m perpendicular away from the rail axis. The coordinate system, the position of the lasertracker and the rail axis (pointing in the direction of the *x*-axis) are depicted in the left part of Figure 10. The uncertainties $u_{x}$, $u_{y}$ and $u_{z}$ for the three axes *X*, *Y* and *Z* are shown separately in the midst of Figure 10. For the evaluation of the quality of the point accuracy, the scalar value of the three uncertainties $u_{P}$ can be calculated with Equation (7):

(7) $$u_{P}=\sqrt{u_{x}^{2}+u_{y}^{2}+u_{z}^{2}}$$

This average point error is known as Helmert’s point error. The plot on the right of Figure 10 depicts this point error.

The ISO 12488-1:2012-07 [1] specifies a value of ±5 mm for the dimension “B” of the highest tolerance class, class 1. According to DIN 18710-1:2011-1 [12], the standard deviation $\sigma_{x}$ of a measuring system must be between 10% and 20% of the tolerance of the variable *T* to be determined:

(8) $$0.1\leq \frac{\sigma_{x}}{T}\leq 0.2$$

The measurement uncertainty of the ARTIS system is 0.2 mm (see Figure 10) with respect to the *Y*-axis with a rail length of 110 m (eccentric measurement). The requirement is thus fulfilled.

A tolerance of 1 mm is required for the dimension “b” of the same directive and the same tolerance class. This measure is the arrow height relative to a base of 2 m. The requirement is also satisfied with 0.2 mm as shown in Figure 10 and Equation (8).

### 4.2. Measurement Uncertainty of the Burr Width at the Rail Head

**Figure 11:**
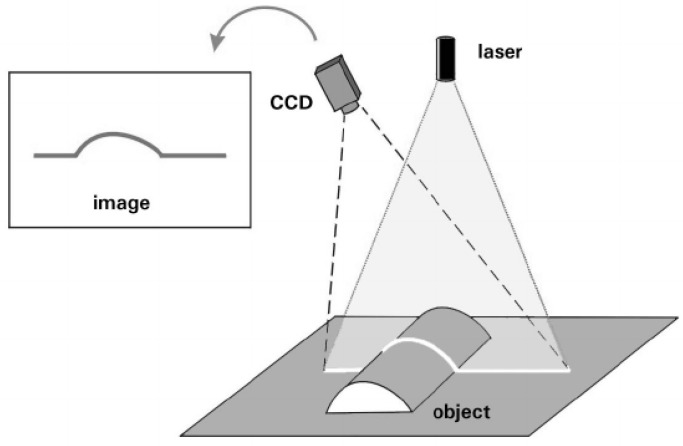
The principle of the light-section method is a 3D method for the noncontact measurement of a profile in an intersecting plane. According to the principle of the optical triangulation a collimated (parallel light) laser beam is emitted, reflected on the surface of the target, and projected and stored on a charge-coupled device (CCD). The position of the laser and camera, as well as the angle between them, must be known (through calibration). The spatial position of the target points can be calculated by triangulation. The position of the target points can be calculated with subpixel accuracy. A 3D image is obtained by the scanning and dense parallel succession of profile sections in the direction of scanner movement [13]. (Reproduced with permission from Mrs. Raffaella Fontana, SPIE; published by Renzo Salimben, 2003.)

Two PLSs are used to determine the target values. Figure 11 explains the measurement principle of the light-section method. Transversely to the direction of travel, the PLS measure 640 points per profile with a frequency of 100 Hz. With an average measuring range of 100 mm, the point distance results in 100 mm/639 ≈ 0.16 mm. Regarding the height (*Z* axis), the sensors have a measurement uncertainty of 0.16 mm(1*σ*) at a standard measuring range of 240 mm. The smallest detectable change in height is 12 μm.

**Figure 12:**
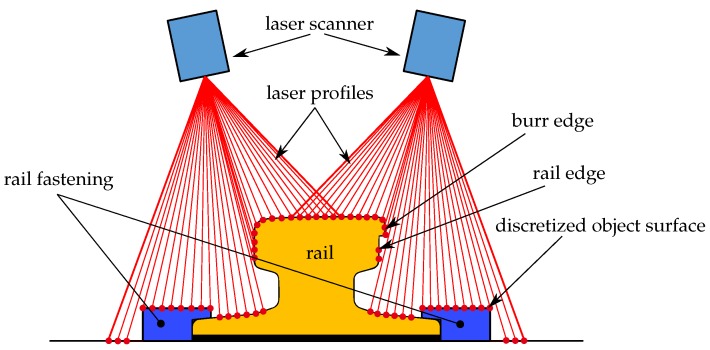
Schematic principle of a PLS scanning a worn rail profile and rail fastening.

An example is the determination of the measurement uncertainty of the burr width by a PLS. The geometrical context is shown in Figure 12. The functional relationship is described by Equation (9):

(9) $$\begin{array}{c} Y_{burr}=f\left(Y_{burr\_edge},Y_{rail\_edge}\right)=Y_{burr\_edge}-Y_{rail\_edge} \end{array}$$

Table 3 shows the influencing variables and measurement uncertainties for the determination of the burr. Analogous to the example depicted in Section 4.1 both uncertainties of type B were quantified by means of information provided by the company and the distributions are assumed to conform with the normal distribution.

The measurement uncertainty of the *Y* component of the burr is calculated by setting up the partial derivatives with respect to the influencing values (see Equation (5)).

By inserting them into the uncertainty–propagation law, we obtain:

(10) $$u_{Y_{burr}}^{2}=u_{Y_{edge\_burr}}^{2}+u_{Y_{edge\_rail}}^{2}$$

The calculation of the measurement uncertainty of the burr results in:

$$u_{burr}=0.23\;\mathrm{mm}$$

The calculated measurement uncertainty for the determination of the burr is significantly smaller than the required millimeter and thus can be regarded as sufficient.

## 5. Hardware and Software

### 5.1. Hardware

The acquisition of the defined target values (Section 2) imposes some requirements on the mechanical platform, which are discussed in the following section.

A platform with low weight had to be developed in order to enable transport by aircraft. At the same time, it must be very rigid in order to prevent increased noise in the measurements induced by deformation. The task was solved by using lightweight materials and a sandwich-structured composite for the vehicle platform.

The modular structure has already been described in Section 3. For feasibility it is necessary that PLSs and cameras are easy to dismantle. A centering pin is used, which ensures high repeatability and simplicity in operation. The latter applies not only to the mechanical system, but also to the electronics: connection and disconnection are achieved with little effort by means of a contact strip.

For an optimal determination of the target values, the position of the PLS and cameras must be variable. Two pairs of a PLS and a camera, each firmly connected, are arranged on a bar in a rotatable and displaceable manner. By alignment to the current rail profile an optimal perspective can be ensured.

Since the vehicle moves independently on a profile and as cables are not suitable for power supply and data transfer, the battery capacity has to guarantee autonomous operation as long as possible. To achieve this the sensors can be optionally switched on and off. For a continuous and consistent propulsion the vehicle is equipped with two electric engines.

**Figure 13:**
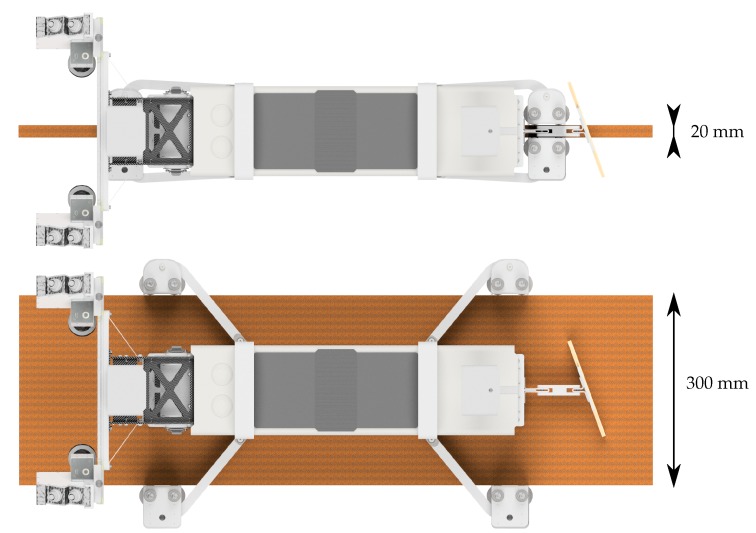
Minimal (top) and maximal (bottom) position of the adaptable guidance system.

In order to cover a wide range of profiles, an adaptable guidance system was developed. This allows one to drive along profiles with head or flange widths between 20 and 300 mm (see Figure 13). The availability is enhanced by a single-sided suspension system and by height-adjustable guide rollers. In case of changing head widths the gap between the guide rollers can be adjusted via remote control. The deliberately small cross-section is not restricted by the adjustable guidance system. The vehicle can also be used in narrow spaces. The risk of a collision with adjacent building components is minimized.

In order to always ensure an optimal angle of incidence on the CCR, the bracket of the CCR can rotate around the vertical and the tilting axis for an improvement of the work process. While moving the orientation of the CCR is automatically adjusted to ensure the optimal angle of incidence to the surveying instrument. The use of a 360° prism instead of the CCR was discarded due its low accuracy (centering accuracy: 2 mm) [7].

### 5.2. Electronic Components

**Figure 14:**
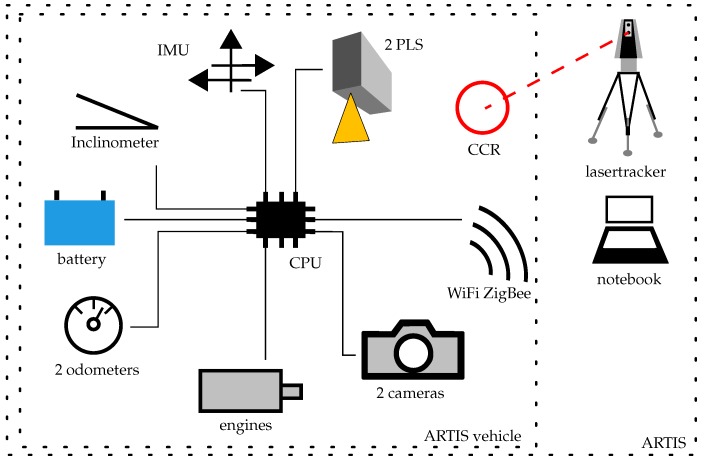
Electronic components of ARTIS.

In the following section the electronic components of ARTIS are presented (Figure 14). A single-board computer (central processing unit, CPU) serves as a computing unit on the vehicle. It controls the modules and stores the data. Furthermore it communicates with the operating and evaluation unit, located on a notebook, by receiving, processing and replying commands.

By using extensible electronic components, the various sensors can be controlled and the data can be merged.

The used components extend the measuring system through an IMU, a temperature sensor, a controller for the motors and the adaptable guidance system, the control of the rotating unit, the information on the power consumption and a module for switching the components (e.g., the PLS, cameras, lighting, etc.) on and off.

For measuring the inclination, the data of an high-precision inclinometer as well as the data of an IMU are used. The IMU is used for an additional task. If rail joints are welded and the head is ground, the detection by means of the PLS or camera images is difficult. Due to increased accelerations during an overpass, the IMU is used. It measures with a frequency of 100 Hz. In ARTIS a microelectromechanical system (MEMS)-based low-cost variant is completely sufficient for the task.

Twelve-volt motors are used to drive the vehicle and to move the guidance system. Two encoders measure the traveled way in autonomous operation.

Two servos turn the rotary unit, which accommodates the CCR.

The two industrial cameras are used to support the measurement results of the PLS. These are mounted parallel to the PLS on the vehicle. With the universal serial bus (USB) port the data is transported to the data storage unit. Pictures can be taken with a resolution of 5 megapixels. Due to the calibration and a trigger signal, the images are georeferenced and can therefore be used to evaluate the measurement results of the PLS.

The two PLSs also have trigger inputs and network interfaces for storing the data.

ZigBee and WiFi modules are used for the radio connection between the operating and evaluation unit on the notebook and the computing unit on the vehicle.

### 5.3. Implementation

The design was realized by a 3D computer-aided design (CAD) software. Besides the arrangement of the electronic components, cable routing was planned due to the very little installation space. Wherever reasonable and possible, purchased parts were designated and utilized during the design phase. The frequently available 3D models of these parts, provided by the suppliers, accelerated the construction time. Furthermore purchased parts are usually cheaper, optimally designed, available at short notice and documented by means of data sheets.

For a special design, not all components can be purchased. Applying a tailored design, lasered and possibly edged sheet metal parts, made of aluminum or stainless steel, were an appropriate alternative to milled metal components.

Three-dimensional printed components, consisting of a variety of materials, were also taken into account during the design.

### 5.4. Software

**Figure 15:**
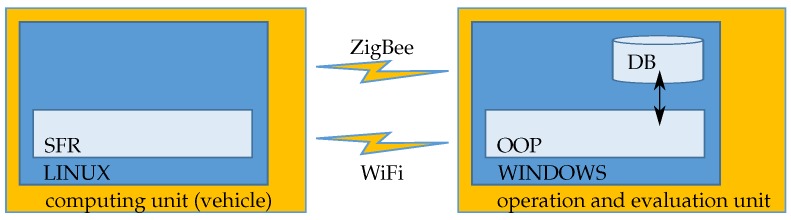
System architecture of the software of ARTIS. SFR: software framework for robots; OOP: object-oriented programming; DB: database.

The system architecture of ARTIS encloses the software on the computing unit, located on the vehicle, and the software on the operating and evaluation unit, located on a notebook controlled by the operator (Figure 15).

A Linux operating system runs on the computing unit of the vehicle. Based on this, a software framework for robots (SFR) is installed. All sensors are controlled via the SFR, and the data is stored on a storage device connected to the computing unit.

The operating and evaluation unit is located on a notebook with a Microsoft Windows operating system. The program is written in an object-oriented programming language (OOP). The computing unit and the operating and evaluation unit communicate via two wireless connections. The data is stored in a database (DB).

## 6. Calibration of the PLS

### 6.1. Superior Vehicle Coordinate System

**Figure 16:**
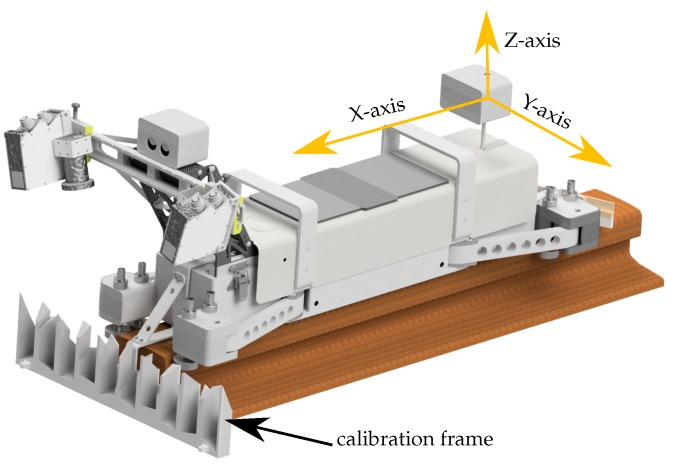
Origin of the vehicle coordinate system (VCS).

The basis for a common analysis of all the sensors, which are located on the vehicle is a common coordinate system. For this purpose, a vehicle coordinate system (VCS), in literature also known as body frame, was introduced, to which the positions of the sensors relate (Figure 16). In order to realize and repeatably recover the VCS, the vehicle contains several bores, into which 0.5 inch corner cube reflectors can be placed. For the calibration, the bores are used to transform the calibration frame into the VCS (see Section 6.3 and Section 6.4). Furthermore they are used to control the stiffness of the vehicle.

### 6.2. Basic Idea

In order to fulfill the ambitious requirements regarding the accuracy of the target values (see Table 1), the system has to be calibrated with high accuracy. The position ($t_{x},t_{y},t_{z}$) and the orientation (ω,ϕ,κ) of the PLS (also known as six degrees of freedom (6DoF)) in the VCS have to be determined with an accuracy of 0.1 mm in position and 0.1° in orientation. It is not possible to directly measure the decisive 6DoF with a high accuracy. Thus the calibration has to be realized via the object space. In [14] a general model to solve the calibration with certain planes of a calibration frame in the object space is introduced. These (altogether *s*) planes of the calibration frame are measured with an external sensor with superior accuracy as well as with the sensor which has to be calibrated. The measurements of the external sensor are used to determine plane parameters in Hesse normal form (normal $n={\left[n_{x,i},n_{y,i},n_{z,i}\right]}^{T}$ and distance parameter $d_{i}$) for each plane *i* (with i∈1,...,s) of the calibration frame. By transforming these plane parameters into the VCS the functional model of the adjustment consists of the following equations:

(11) $$\begin{array}{c} X_{VCS,j}=\left[\begin{array}{c} x_{VCS,j}\\ y_{VCS,j}\\ z_{VCS,j} \end{array}\right]=\left[\begin{array}{c} t_{x}\\ t_{y}\\ t_{z} \end{array}\right]+R_{x}\left(\omega \right)R_{y}\left(\phi \right)R_{z}\left(\kappa \right)\left[\begin{array}{c} x_{PLS,j}\\ y_{PLS,j}\\ z_{PLS,j} \end{array}\right] \end{array}$$

(12) $$\begin{array}{c} d_{j}=X_{VCS,j}^{T}n_{VCS,i}-d_{VCS,i} \end{array}$$

$X_{PLS,j}={\left[x_{PLS,j},y_{PLS,j},z_{PLS,j}\right]}^{T}$ represents the *j*-th cartesian coordinate (in the local coordinate system of the PLS) resulting from a measurement of the PLS to plane *i* of the calibration frame. This cartesian coordinate is transformed into the VCS by means of the 6DoF ($t_{x},t_{y},t_{z},\omega ,\phi ,\kappa$) and stored in $X_{VCS,j}$. $n_{VCS,i}$ and $d_{VCS,i}$ contain the plane parameters (in the VCS) of plane *i*. Through inserting Equation (11) in Equation (12) the functional relation is complete. By solving the resulting nonlinear Gauss–Helmert model (GHM, in the sense of [15], p. 416) the distance vector $d={\left[d_{1},...,d_{j},..,d_{r}\right]}^{T}$ (where *r* equals the total number of measurements of the PLS to the calibration frame) is minimized and the desired 6DoF can be obtained. Hartmann et al. [16] describe this calibration method in detail and apply it to a related kinematic multi sensor system.

### 6.3. Calibration Frame

The complexity with the system presented in this paper is created by the fact that the PLS and cameras are movable and can be adjusted in plenty of reasonable set-ups. For all these set-ups the calibration parameters have to be determined with the required accuracy. In order to fulfill this task the accuracy of the calibration parameters for different shapes of the calibration frame was calculated by variance propagation and Monte Carlo simulation, using the functional relationships in Equations (11) and (12). By systematic testing of different shapes and constellations the optimal shape of the calibration frame was determined. Figure 16 depicts the developed calibration frame, which makes it possible to determine the position and orientation with an accuracy of 0.1 mm for the position and 0.1° for the orientation, respectively, in every reasonable set-up of the PLS and cameras. Altogether the calibration frame consists of 61 (=s) measurable, tilted and shifted planes, which were manufactured via 3D printing of grey synthetic material. The calibration frame can be attached to the vehicle by using centering pins. This ensures high repeatability and simplicity in operation. The planes of the calibration frame were measured previously and only once, using a lasertracker in combination with a high accurate scanning system (Leica T-Scan, Leica Geosystems AG, Heerbrugg, Switzerland, [17]). The plane parameters were derived from the resulting point cloud. By additionally measuring the positions of the bores on the vehicle with the lasertracker the plane parameters could easily be transformed into the VCS.

### 6.4. Workflow

**Figure 17:**
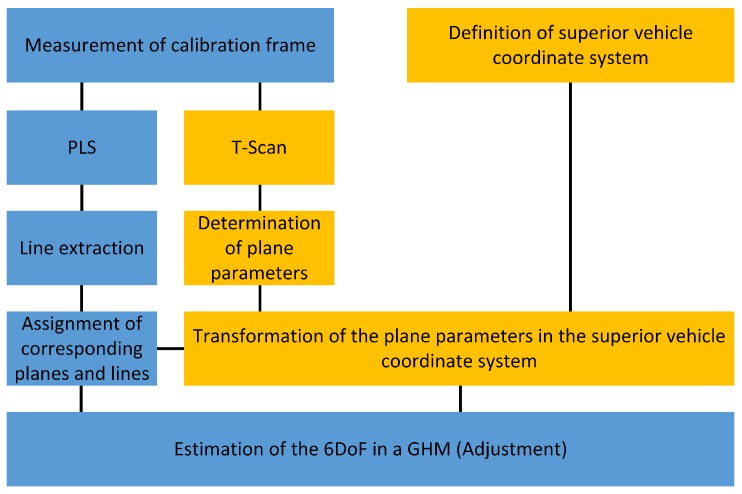
Workflow of the calibration process (following [14]). 6DoF: six degrees of freedom; GHM: Gauss-Helmert model.

The workflow of the calibration process is depicted in Figure 17. The yellow shaded tasks have to be executed once respectively for reasons of control at suitable intervals. They are already described in Section 6.1 and Section 6.3.

The blue shaded tasks have to be executed before measuring with a new set-up, which is the case when a PLS is shifted or turned. In order to accelerate the calibration process these tasks proceed automatically.

After attaching the calibration frame to the vehicle both PLS measure on the calibration frame. This task is followed by a subsequent elimination of data points not lying on the calibration frame.

In the task “Line extraction” the measured data is divided in straight lines by using the Ramers–Douglas–Peucker algorithm.

The following task contains the assignment of the extracted straight lines to the planes of the calibration frame. This assignment is accomplished by improving the approximate values for the 6DoF by means of the PLS data. The improved approximate values were used to rotate and shift the PLS data, and finally the assignment is chosen, for which the squared distance between planes and PLS data is minimal.

The final adjustment comprises the exact estimation of the 6DoF by applying the method of [14] through solving the GHM described in Section 6.2.

After the calibration process the calibration frame is removed from the vehicle and the system is usable.

## 7. Results

**Figure 18:**
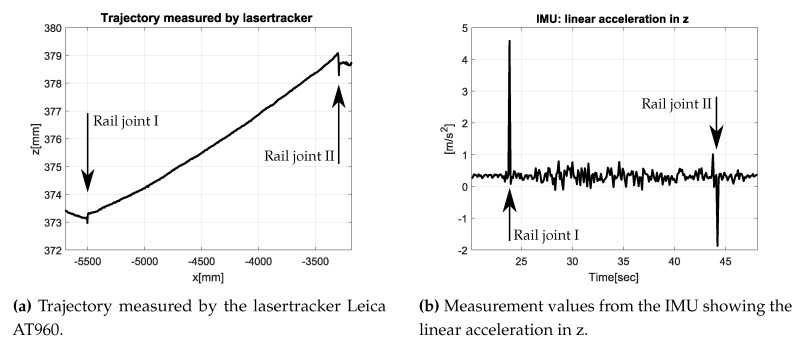
Results from lasertracker and IMU measurements.

In this section the results of test measurements are presented. Figure 18 depicts the measurements of the tracked ARTIS vehicle, running on an about a 6-m-long rail, consisting of three rails of equal length. The rail joints are not welded and have a gap of about 10 mm. Figure 18a comprises a part of the trajectory in *xz*-plane. The rail joints can be clearly identified by an abrupt vertical movement. Additionally the rail joints are detected in the data of the IMU. In Figure 18b the linear acceleration in *z*-direction of the same trajectory is visualized.

**Figure 19:**
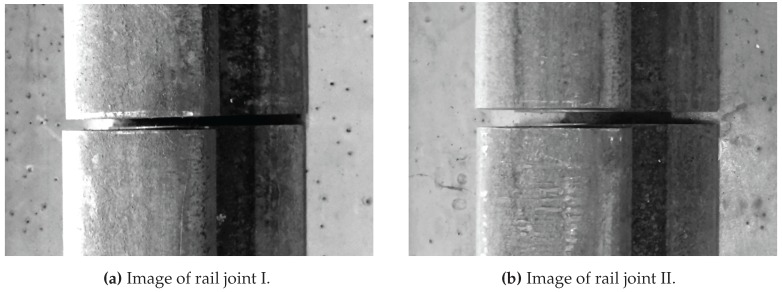
Images of rail joints on the test track.

Figure 19 shows the two rail joints on the test track. The images were captured by one camera of the ARTIS vehicle during the test measurements.

**Figure 20:**
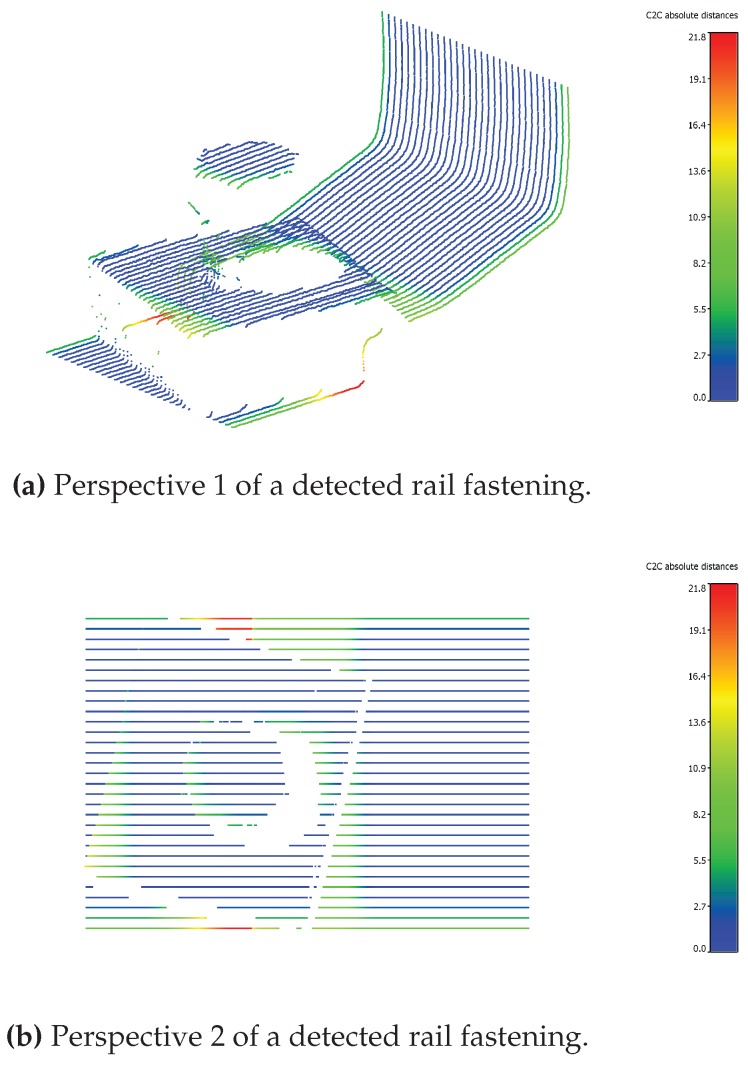
Two perspectives of a comparison between a measured rail fastening and a correct positioned rail fastening.

Figure 20 shows a point cloud of a part of a rail, inclusive rail fastening, captured by one of the PLS. The data is compared to a correct positioned fastening, measured beforehand. The point cloud is colored by the cloud to cloud distance between the two fastenings. As can be seen the fastening is not in the correct position, but rotated.

## 8. Conclusions

The procedures for measuring crane runways have been continually refined. The last major development step was the introduction of self-propelled rail vehicles whose position can be determined by kinematic surveying. The Advanced Rail Track Inspection System (ARTIS) extends the possibilities with additional sensors. This makes it possible to determine target values which so far could not be determined or could only be determined with a great effort. Thus, in addition to the established target values, such as the position and height of the rail, it is also possible to detect the rail profile itself and its rail fastening. The provided information enables an objective assessment of the condition of a crane runway and is the basis for further activities, e.g., maintenance work. It has been demonstrated that the used sensors can determine the target variables with sufficient accuracy. The vehicle platform takes the various requirements of size, weight and variability into account. The procedure for the calibration of the profile laser scanner (PLS) was presented. Only through this a large part of the new target values can be determined.

## Figures and Tables

**Table 1:** Target values of the survey with ARTIS.

Target Value	Coordinate	Reference	Accuracy	Sensors
position of the rails	*X*, *Y*	absolute	submillimeter	lasertracker, optional PLS
height of the rails	*X*, *Z*			
position and height of adjacent rails	*X*, *Y*, *Z*			
longitudinal inclination of the rail head	*X*, *Z*	absolute	<0.06°	lasertracker, inclinometer
lateral inclination of the rail head	*Y*, *Z*		<0.01°	
practical and theoretical rail axes	*X*, *Y*, *Z*	absolute	submillimeter	lasertracker, PLS
breaking out in the rail head	*X*, *Y*, *Z*	absolute, relative	centimeter (*X*), submillimeter (*Y*, *Z*)	lasertracker or odometer, PLS, camera
wheel slip marks				
burr on the rail head				
long and transverse cracks in the rail head				
rail height	*X*, *Z*	absolute, relative	centimeter (*X*), submillimeter (*Z*)	lasertracker or odometer, PLS
position of rail fastenings	*X*	absolute	millimeter	lasertracker or odometer, PLS, camera
condition of rail fastenings				
position of the rail joint				lasertracker or odometer, PLS, camera, IMU
type of a rail joint				

**Table 2:** Significant influencing values for determining the position of the *Y*-axis of the ARTIS vehicle.

Influencing Value		Uncertainty	Type
$Y_{P}$	*Y* component of the position of the lasertracker	$u_{Y_{P}}=0.026\;\mathrm{mm}$	A
*z*	zenith / elevation angle	$u_{afix}=8.7\;\mu m,u_{avar}=3.5\;\mu m/m$	B
$S_{d}$	slope distance	$u_{dfix}=5.8\;\mu m,u_{dvar}=0.3\;\mu m/m$	B
*t*	azimuth	$u_{afix}=8.7\;\mu m,u_{avar}=3.5\;\mu m/m$	B
$Y_{gauge\_line}$	*Y* component offset target point to gauge line	$u_{gauge\_line}=0.01\;\mathrm{mm}$	A
rot_dev	centering error rotating device	$u_{rot\_dev}=0.01\;\mathrm{mm}$	A
$CCR_{EC}$	CCR error center	$u_{CCR_{EC}}=0.006\;\mathrm{mm}$	B
$CCR_{ESS}$	CCR error spherical shape	$u_{CCR_{ESS}}=0.0015\;\mathrm{mm}$	B
$Z_{CCR-ToR}$	*Z* component offset CCR-top of rail	$u_{Z_{CCR-ToR}}=0.01\;\mathrm{mm}$	A
in	inclination from inclinometer	$u_{in}=0.01\;\mathrm{gon}$	B

**Table 3:** Significant influencing values for the determination of the burr.

Influencing Value		Uncertainty	Type
$Y_{edge\_burr}$	edge burr	$u_{Y_{edge\_burr}}=0.16\;\mathrm{mm}$	B
$Y_{edge\_rail}$	edge rail	$u_{Y_{edge\_rail}}=0.16\;\mathrm{mm}$	

## Abbreviations

The following abbreviations are used in this manuscript:

ARTIS	Advanced Railtrack Inspection System
CAD	Computer-aided design
CCD	Charge-coupled device
CCR	Corner cube retroreflector
CPU	Central processing unit
CTSS	Crane track surveying system
DB	Database
GHM	Gauss-Helmert model
GUM	Guide to the Expression of Uncertainty in Measurement
IMU	Inertial measurement unit
ISO	International Organization for Standardization
lre	Left rail edge
MEMS	Microelectromechanical system
OOP	Object-oriented programming
PLS	Profile laser scanner
rre	Right rail edge
6DoF	Six degrees of freedom
SFR	Software framework for robots
USB	Universal serial bus
VCS	Vehicle coordinate system
wlre	Worn left rail edge
wrre	Worn right rail edge
